# Healthcare Resource Utilization and Cost Comparisons of High-Protein Enteral Nutrition Formulas Used in Critically Ill Patients

**DOI:** 10.36469/001c.36287

**Published:** 2022-07-01

**Authors:** Matthew C. Bozeman, Laura L. Schott, Amarsinh M. Desai, Mary K. Miranowski, Dorothy L. Baumer, Cynthia C. Lowen, Zhun Cao, Krysmaru Araujo Torres

**Affiliations:** 1 University of Louisville Health, Louisville, Kentucky; 2 PINC AI™ Applied Sciences, Premier Inc, Charlotte, North Carolina; 3 Nestlé Health Science, Bridgewater Township, New Jersey

**Keywords:** L-arginine, immunonutrition, enteral nutrition, healthcare resource utilization, critical care, high protein, surgery

## Abstract

**Background:** High-protein enteral nutrition is advised for patients who are critically ill. Options include immunonutrition formulas of various compositions and standard high-protein formulas (StdHP). Additional research is needed on the health economic value of immunonutrition in a broad cohort of severely ill hospitalized patients.

**Objective:** The study goal was to compare healthcare resource utilization (HCRU) and cost between immunonutrition and StdHP using real-world evidence from a large US administrative database.

**Methods:** A retrospective cohort study was designed using the PINC AI™ Healthcare Database from 2015 to 2019. IMPACT® Peptide 1.5 (IP) was compared with Pivot® 1.5 (PC), and StdHP formulas. Inclusion criteria comprised patients age 18+ with at least 1 day’s stay in the intensive care unit (ICU) and at least 3 out of 5 consecutive days of enteral nutrition. Pairwise comparisons of demographics, clinical characteristics, HCRU, and costs were conducted between groups. Multivariable regression was used to assess total hospital cost per day associated with enteral nutrition cohort.

**Results:** A total of 5752 patients were identified across 27 hospitals. Overall, a median 7 days of enteral nutrition was received over a 16-day hospital and 10-day ICU stay. Median total and daily hospital costs were lower for IP vs PC ($71 196 vs $80 696, *P*<.001) and ($4208 vs $4373, *P*=.019), with each higher than StdHP. However, after controlling for covariates such as mortality risk, surgery, and discharge disposition, average total hospital cost per day associated with IP use was 24% lower than PC, and 12% lower than StdHP (*P*<.001). Readmissions within 30 days were less frequent for patients receiving IP compared with PC (*P*<.02) and StdHP (*P*<.001).

**Discussion:** Choice of high-protein enteral nutrition for patients in the ICU has implications for HCRU and daily hospital costs. Considering these correlations is important when comparing formula ingredients and per unit costs. Among the enteral nutrition products studied, IP emerged as the most cost-saving option, with lower adjusted hospital cost per day than PC or StdHP.

**Conclusions:** Using a select immunonutrition formula for critically ill patients may provide overall cost savings for the healthcare system.

## BACKGROUND

Critical care nutrition guidelines from the American Society for Parenteral and Enteral Nutrition (ASPEN) and the Society of Critical Care Medicine (SCCM) advise providing an increased amount of protein for patients hospitalized in intensive care units (ICU), and when volitional intake is not possible, also suggest enteral nutrition (EN) formulas containing supplemental immunonutrients for surgical and trauma patients.^1,2^ High-protein (22%-25% of calories) EN comes in standard form or with various combinations of immunonutrients. Further, sources and amounts of macronutrients vary, as do micronutrient levels when comparing high-protein EN.

Guidelines suggest routine use of postoperative immunonutrition (IM) formula in the surgical ICU and largely reference randomized controlled trial data from patients who had major elective surgery. These patients had decreased infectious complications and length of stay (LOS) after receiving high-protein formula containing supplemental L-arginine, ω-3 fatty acids, and nucleotides.^2,3^ Recent meta-analyses and reviews support arginine containing IM vs standard formulas for trauma and surgical ICU patients.^1,4–6^ Real-world evidence has also shown perioperative use of formulas containing these immunonutrients reduced complications and LOS in head and neck cancer surgery.^7^ Additionally, volume-based feeding in surgical trauma patients in the ICU with formula containing this combination of immunonutrients was associated with improved protein adequacy, better blood glucose management, and decreased pneumonia compared with rate-based feeding of standard formula.^8^ Nonetheless, populations of critically ill patients are heterogenous, and management of nutritional support in patients who are critically ill continues to be the subject of debate.^9^

Despite the guidelines and existing evidence, large numbers of surgical ICU patients receive lower-cost, standard high-protein (StdHP) enteral feedings that do not contain added immunonutrients. In addition, many other patients in the ICU receive IM formulas that have various degrees of substantiation in the literature. Studies relating EN choice to the cost of acute hospitalization are sparse, and an evidence gap exists for comparing IM formulas.^10–12^

Recommendations for cost-effective clinical strategies and value assessment of healthcare suggest that multiple factors, such as severity of disease and associated negative aspects, should be considered when evaluating use of healthcare products and technologies.^13,14^ These recommendations are supported by the ASPEN/SCCM guidelines, which suggest assessment of nutrition risk, comorbid conditions, and tolerance.^1^ A review and model by Tyler et al^15^ demonstrated that optimizing nutrition support therapy could save Medicare millions of dollars through shorter hospital stays and complication avoidance. The total projected cost savings of $580 million dollars was reported for 5 therapeutic areas: sepsis ($222 million), gastrointestinal cancer ($242 million), hospital-acquired infections ($85 million), surgical complications ($33 million), and pancreatitis ($2 million). A quality improvement program targeting nutrition intervention for malnourished hospitalized patients also showed reduced LOS and infection rates.^16^ Of note, patients having surgery for colorectal cancer or aortic aneurysm in this study received IM.

Providing the most evidence-based, high-quality hospital care at the lowest cost requires the comparison of outcomes and costs for high-protein IM formulas and StdHP formulas. Further, measurement for association between hospital cost and choice of high-protein EN used in the ICU is needed.

### Objectives

With this in mind, we utilized real-world data from an extensive database encompassing multiple US hospitals to retrospectively describe adults having an ICU stay and EN formula use. We assessed 3 groups: 2 groups exclusively using different IM formulas, and 1 group exclusively using StdHP formulas. Thereby, the objective of the study was to compare healthcare resource utilization (HCRU) and cost across these EN cohorts and identify the cost-saving option. Further, we used a large database in which multiple measures of comorbidity, resource utilization, and patient and visit characteristics could be examined with LOS and cost.

## METHODS

### Data Source and Study Design

A retrospective cohort study examining characteristics of patients receiving high-protein (≥25% kcal) EN was conducted using data from the PINC AI™ Healthcare Database (PHD; formerly, Premier Healthcare Database). The sample consisted of eligible inpatient encounters admitted from October 1, 2015, through February 28, 2019, age 18 years and older, with a minimum of 1 billed day of ICU utilization (identified via room and board charges billed to an ICU or critical care unit), and with a billing record of at least 3 days of consecutive use or 3 days of use within 5 consecutive days of EN (Table 1) as grouped: IMPACT® Peptide 1.5 (IP), Pivot® 1.5 Cal (PC), and StdHP limited to Promote®, Promote® with Fiber, Replete®, and Replete® Fiber. The 3 EN groups were mutually exclusive, and patients with crossovers during hospitalization were excluded. The PHD is a large, hospital-based database containing billing and service information from inpatient and hospital-based outpatient visits from hospitals across the United States. The PHD captures about 25% of all US inpatient hospital discharges and averages more than 8.5 million inpatient and 75 million outpatient encounters per year. Patient data can be tracked within a hospital system through a unique PHD patient key. Institutional review board approval for this study was not required, based on 45 CFR § 46, because the study used existing de-identified hospital discharge data, and recorded information could not be identified directly or through identifiers linked to individuals. All data were compliant with the Health Insurance Portability and Accountability Act (HIPAA), and informed consent was not pursued.

**Table 1.** Nutrition Comparison of the EN Groups Studied,<sup>a</sup> per Liter

**EN Formula Contents**	**IP**	**PC**	**StdHP**
Kcal/mL	1.5	1.5	1.0
Protein, g (%)	94 (25%)	93.8 (25%)	64 (25%)
Source	Hydrolyzed casein and arginine	Hydrolyzed casein, whey, and arginine	Soy and casein
Supplemental arginine (g)	18.7	11	—
Total arginine (g)	20.8	13	Inherent to soy and casein
Carbohydrate, g (%)	140 (38)	172.4 (45)	112, 124 (45)/130, 138.3 (52, 50)
Fiber (g)	—	7.5	+/-12/14
Fat g (%)	63.6 (37)	51 (31)	34 (30)/26, 28.5 (23, 25)
Ω6:Ω3	1.5:1	1.7:1	2.3, 2.4:1/9.8:1
EPAg + DHA (g)	4.9	3.7	—
MCT:LCT	50:50	20:80	20:80/19:81
MCT (g)	31.8	10.2	6.8/4.9, 5.4
Nucleotides (g)	1.8	—	—
Select micronutrients			
Vitamin C (mg)	1000	304	200/346, 342
Vitamin E (mg)	68	27	20/25, 25.3
Copper (mg)	3	2.2	1.6/2.0
Manganese (mg)	4	5.1	3.6/5.1
Selenium (mcg)	100	80	68, 60/72
Zinc (mg)	36	30.8	16/241

Abbreviations: DHA, docosahexaenoic acid; EPA, eicosapentaenoic acid; IP, IMPACT® Peptide 1.5; LCT, long-chain triglycerides; MCT, medium-chain triglycerides; PC, Pivot® 1.5 Cal; StdHP, Replete®/Promote® Ω6:Ω3, ratio of omega-6 and omega-3 fatty acids. ^a^IMPACT® and Replete® are registered trademarks of Société des Produits Nestlé S.A.; Pivot® and Promote® are registered trademarks of Abbott Laboratories.

### Patient Demographics, Visit, and Clinical Characteristics

Data collected included patient age; self-reported sex, race, and ethnicity; primary insurance payer; and comorbid conditions. Severity of illness was assessed via the 3M™ All Patient Refined Diagnosis Related Groups (APR-DRG) Classification System severity-of-illness (SOI) and risk-of-mortality (ROM) measures, Elixhauser index,^17,18^ and Medicare Severity DRG (MS-DRG) extracorporeal membrane oxygenation (ECMO) or tracheostomy procedure. The 30 conditions measured via the Elixhauser index–weighted score were also examined individually (see footnote, Table 2). Visit characteristics included admission type, admission point of origin, and discharge status, which were submitted by hospitals according to the criteria of the Centers for Medicare and Medicaid Services, as defined in the uniform billing instructions. Mechanical ventilation, rectal catheterization, surgery and trauma status, and attending physician specialty were tracked. Hospital characteristics included 2010 US Census geographical region (ie, Midwest, Northeast, South, West), teaching status, urban/rural location, and bed size (ie, 1-199, 200-299, 300-499, or ≥500 beds).

**Table 2.** Patient and Visit Characteristics

**Characteristic**	**Overall (n=5752)**	**IP (n=2525)**	**PC (n=759)**	**StdHP (n=2468)**
Age, years	62 (50, 75)	59 (46, 70)	58 (42, 70)	65 (55, 76)^b^
Age group, years^a^				
18-34	646 (11.2)	357 (14.1)	135 (17.8)	154 (6.2)
35-49	766 (13.3)	380 (15.0)	129 (17.0)	257 (10.4)
50-64	1815 (31.6)	836 (33.1)	224 (29.5)	755 (30.6)
65-79	1842 (32.0)	745 (29.5)	208 (27.4)	889 (36.0)
80+	683 (11.9)	207 (8.2)	63 (8.3)	413 (16.7)
Sex^b^				
Female	2298 (40.0)	866 (34.3)	202 (26.6)	1230 (49.8)
Male	3453 (60.0)	1659 (65.7)	557 (73.4)	1237 (50.1)
Race^b^				
White	4829 (84.0)	2219 (87.9)	606 (79.8)	2004 (81.2)
Black	484 (8.4)	151 (6.0)	93 (12.3)	240 (9.7)
Other/unknown	439 (7.6)	155 (6.1)	60 (7.9)	224 (9.1)
Ethnicity^b^				
Hispanic or Latino	328 (5.7)	71 (2.8)	18 (2.4)	239 (9.7)
Not Hispanic or Latino	4506 (78.3)	1719 (68.1)	705 (92.9)	2082 (84.4)
Other/unknown	918 (16.0)	735 (29.1)	36 (4.7)	147 (6.0)
Healthcare coverage type^b^				
Medicare	2745 (47.7)	977 (38.7)	271 (35.7)	1497 (60.7)
Medicaid	1251 (21.7)	704 (27.9)	138 (18.2)	409 (16.6)
Managed care	565 (9.8)	142 (5.6)	125 (16.5)	298 (12.1)
Commercial	771 (13.4)	505 (20.0)	123 (16.2)	143 (5.8)
Other payer	420 (7.3)	197 (7.8)	102 (13.4)	121 (4.9)
Discharge status^b^				
Inpatient mortality	1043 (18.1)	484 (19.2)	152 (20.0)	407 (16.5)
Home	758 (13.2)	391 (15.5)	65 (8.6)	302 (12.2)
Home healthcare	478 (8.3)	229 (9.1)	48 (6.3)	201 (8.1)
Hospice	285 (5.0)	60 (2.4)	27 (3.6)	198 (8.0)
Intermediate care facility	1268 (22.0)	557 (22.1)	91 (12.0)	620 (25.1)
Other	1920 (33.4)	804 (31.8)	376 (49.5)	740 (30.0)
APR-DRG severity of illness^b^				
Minor/moderate	649 (11.3)	322 (12.8)	98 (12.7)	234 (9.4)
Severe	837 (14.6)	424 (16.8)	71 (9.4)	342 (13.9)
Extreme	4266 (74.2)	1779 (70.5)	592 (78.0)	1895 (76.8)
APR-DRG risk of mortality^c^				
Minor/moderate	954 (16.6)	531 (21.0)	113 (14.9)	310 (12.6)
Severe	1071 (18.6)	469 (18.6)	154 (20.3)	448 (18.2)
Extreme	3727 (64.8)	1525 (60.4)	492 (64.8)	1710 (69.3)
Elixhauser index score	5 (4, 7)	5 (3, 7)	6 (3, 8)^b^	6 (4, 8)^b^
ECMO or tracheostomy	1067 (18.5)	641 (25.4)	192 (25.3)	234 (9.5)^b^
Nausea and vomiting	65 (1.1)	20 (0.8)	6 (0.8)	39 (1.6)a
Diarrhea	268 (4.7)	116 (4.6)	29 (3.8)	123 (5.0)
Abdominal pain	38 (0.7)	8 (0.3)	2 (0.3)	28 (1.1)^b^
Abdominal distention	16 (0.3)	5 (0.2)	7 (0.9)^c^	4 (0.2)
Urinary tract infection	860 (15.0)	335 (13.3)	109 (14.4)	416 (16.9)^b^
*C. difficile* infection	262 (4.6)	116 (4.6)	45 (5.9)	101 (4.1)
Mechanical ventilation	4494 (78.1)	1930 (76.4)	645 (85.0)^b^	1919 (77.8)
Rectal catheterization	470 (8.2)	230 (9.1)	147 (19.4)^b^	93 (3.8)^b^

Abbreviations: APR-DRG, All Patient Refined Diagnosis Related Groups Classification System; ECMO, extracorporeal membrane oxygenation; IP, IMPACT® Peptide 1.5; PC, Pivot® 1.5 Cal; StdHP, standard high-protein tube feeding products. ^a^*P*<.05 for IP vs PC or IP vs StdHP. ^b^*P*<.01 for IP vs PC or IP vs StdHP. ^c^*P*<.001 for IP vs PC and/or IP vs StdHP. ^d^Elixhauser index was assessed using Quan’s algorithm of primary or secondary ICD-10-CM diagnosis codes at discharge. Prevalence of each comorbidity was evaluated, including congestive heart failure, cardiac arrhythmias, valvular disease, pulmonary circulation disorders, peripheral vascular disorders, hypertension, paralysis, other neurological disorders, chronic pulmonary disease, complicated diabetes, uncomplicated diabetes, hypothyroidism, renal failure, liver diseases, peptic ulcer disease excluding bleeding, HIV/AIDS, lymphoma, metastatic cancer, solid tumor without metastasis, rheumatoid arthritis/collagen vascular disease, coagulopathy, obesity, weight loss, fluid and electrolyte disorders, blood loss anemia, deficiency anemia, alcohol abuse, drug abuse, psychosis, and depression.

### Healthcare Resource Utilization and Costs Outcomes

In this study, HCRU was evaluated via LOS, ICU LOS, medication use, nutrition product utilization and tolerance, and hospital readmission. Medication use included antidiarrheal, antiemetic, and antibiotic class drugs. The number of days on these medications was calculated. Nutrition utilization was captured via units of EN billed, days of EN billed, units per day of feeding, and indicators of nutritional intolerance. For discharged patients, all-cause readmission within 30 days was assessed.

Total hospital cost, ICU cost, nutrition cost, total hospital cost per day, and cost associated with medications were used to draw cost comparisons across the 3 EN groups. Total cost was the sum of all costs incurred during hospitalization as reported by the hospital. Since total cost and LOS are typically correlated, these measures were examined as a single variable, total cost per day, which was the primary outcome.

### Statistical Analyses

Pairwise comparisons with IP were done via Wilcoxon rank sum, *t*, and χ^2^ tests. A multivariable generalized linear model with log link and negative binomial variance functions was used to evaluate associations between EN cohort (ie, independent variable) and total cost per day (ie, dependent variable) in this retrospective analysis.

The estimating equation of the regression model was

$$log[E(Y|T,X)]=\beta_{o}+T\beta_{1}+X\beta_{2},$$

where Y is the dependent variable cost per day; T includes the treatment variables (PC and StdHP vs IP, respectively); X includes the covariates; $\beta_{0}$ indicates the intercept term; and $\beta_{1}$, $\beta_{2}$ indicate the regression coefficients.

Prior to model inclusion, variables were evaluated for pairwise differences between EN cohorts, multicollinearity, clinical nutrition relevance, and association with the outcome (ie, total cost per day) with the rationale of controlling for potential confounders without over-parametrizing the model. A priori covariates included patient, hospital, and clinical characteristics, nutrition product utilization, and medication use. (See Figure 3 for complete list of variables.) Sensitivity analyses were completed to assess model fit and properties of variables in the model. For ease of interpretation, regression coefficients were exponentiated, and 95% confidence intervals (CI) were reported. Because of small cell counts, categorical variables were combined as appropriate. All analyses were conducted using SAS version 9.4. Significance was defined at *P*.05.

## RESULTS

### Sample, Hospital, and Visit Characteristics

We identified 5752 patients receiving EN (2525 IP, 759 PC, and 2468 StdHP) across 27 hospitals during the 3.5-year study period. Mean age was 59.8 years (SD, 17.5 years). The sample was primarily male (60.0%), white (84.0%), not Hispanic or Latino (78.3%), and admitted largely from home (72.7%) and nonelectively (90.6%). Consistent with characteristics of the PHD, patients were mainly seen at hospitals in urban areas (85.5%) in the Northeast/South (59.3%), and for this study, patients were frequently treated at teaching hospitals (90.8%) with at least 500 beds (62.5%). Demographic and visit characteristics varied by cohort (Table 2). Approximately one-third of patients receiving IM (ie, IP, PC) had Medicare as their primary insurance payer, compared with nearly two-thirds of patients receiving StdHP. Frequency of discharge to home or home healthcare was higher for patients receiving IP (24.6%) than for patients receiving PC (14.9%) or StdHP (20.4%). Overall, common specialties of attending physicians included critical care surgeons (20.1%), general surgeons (17.6%), internal medicine (17.3%), hospitalists (15.0%), and pulmonary care (7.0%).

### Clinical Characteristics and Comorbidities

Comorbid conditions and indicators of disease type and severity differed across EN cohorts. Overall, 88.8% patients were identified with severe or extreme APR-DRG SOI, and 83.4% patients were identified with severe or extreme APR-DRG ROM. However, there were significant differences in distributions of SOI and ROM when PC and StdHP were compared with IP (Table 2). Medicare Severity DRG coding of ECMO or tracheostomy was comparable for the IM groups (25%) but only registered 9% for the StdHP patients. Across all groups, patients with this code primarily had tracheostomy procedures, and fewer than 1% received ECMO.

**Figure 1.**
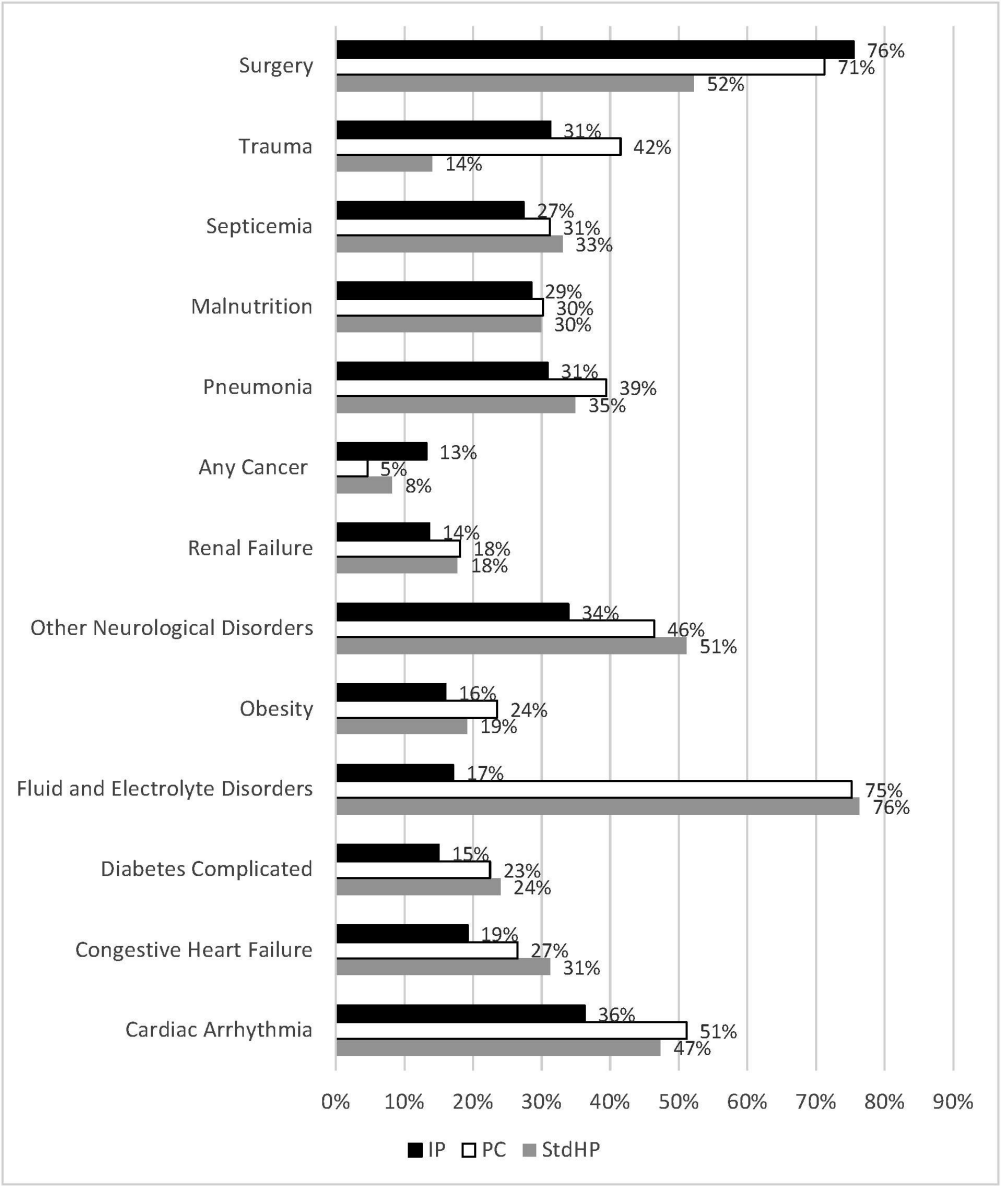
Select Clinical Characteristics and Diagnoses by EN Group^a^ Abbreviations: IP, IMPACT® Peptide 1.5; PC, Pivot® 1.5 Cal; StdHP, standard high-protein tube feeding products. ^a^*P*.05 for IP vs PC and IP vs StdHP, except malnutrition.

Overall, median Elixhauser score was 5, with the most common conditions being fluid and electrolyte disorders (73.8%) and hypertension (61.5%). The frequency of several comorbidities, including complicated diabetes, obesity, septicemia, pneumonia, and urinary tract infection, was lower in the IP cohort (Figure 1) compared with the other EN cohorts. In contrast, frequency of cancer and surgery diagnoses was significantly greater in patients who received IP compared with PC and compared with StdHP. Nearly a third of patients were diagnosed with malnutrition, regardless of EN cohort. Use of mechanical ventilation was reported less frequently in patients receiving IP compared with PC (76.4% vs 85.0%, *P*.001), but use did not differ between IP and StdHP.

### Healthcare Resource Utilization

**Nutrition product use and tolerance**: Product use differed by EN cohort, although across cohorts the treatment pattern was at least 3 consecutive days for approximately 90% of patients (Table 3). Median days of feeding across groups was 7 days. This did not differ for IP and PC groups; however, the StdHP group was billed for a median of 6 days of feeding (*P*.001 vs IP). Patients who received IP had a greater median volume of 1L units billed, compared with PC, (9 vs 8, *P*=.002) and StdHP, (9 vs 6, *P*.001). Similarly, patients who received IP had higher median units per day of feeding compared with patients receiving PC (1.20 vs 1.17, *P*.001) and StdHP (1.20 vs 1.07, *P*.001), respectively. Indicators of EN intolerance, eg, nausea and vomiting, abdominal pain and distension, and diarrhea, were each coded in less than 5% of the sample.

**Table 3.** Healthcare Resource Utilization by EN Group

**Characteristic**	**Total (n=5752)**	**IP (n=2525)**	**PC (n=759)**	**StdHP (n=2468)**
Nutrition pattern of EN for 3 consecutive days (vs EN for 3 days in 5)	5179 (90.0)	2237 (88.6)	701 (92.4)^a^	2241 (90.8)^b^
Nutrition utilization				
Days of EN use	7 (4, 11)	7 (4, 11)	7 (5, 12)	6 (4, 10)^c^
EN cost ($)	79 (25,188)	$109 (9, 240)	248 (132, 422)^c^	43 (21, 83)^c^
1000 mL units of EN billed	7 (4, 13)	9 (5, 15)	8 (5, 14)^a^	6 (3, 10)^c^
EN units billed per day	1.15 (1.00, 1.36)	1.20 (1.00, 1.50)	1.17 (1.00, 1.29)^c^	1.07 (0.50, 1.33)^c^
LOS	20.6 (16.9)	21.9 (18.3)	22.2 (17.3)	18.8 (15.1)^c^
ICU LOS	12.2 (10.0)	12.7 (11.0)	15.3 (11.1)^c^	10.9 (8.1)
Total cost per day ($)	4407 (2127)	4654 (2263)	4821 (2257)	4028 (1867)^c^
Cost of ICU stay ($)	28 208 (29 189)	33 015 (37 678)	30 734 (30 734)^b^	22 514 (17 959)^c^
Total medication cost ($)	493 (176, 1183)	591 (201, 1452)	600 (222, 1311)	416 (143, 958)^c^
Percent of patients with antibiotic use	94.4	94.2	97.1	93.8
Days of antibiotic use	18 (12, 27)	19 (12, 29)	19 (13, 29)	17 (12, 25)^c^
Percent of patients with antidiarrheal medication use	19.5	26.7	15.7	13.4
Days of antidiarrheal medication use	7 (3, 14)	9 (5, 16)	3 (2, 9)^c^	5 (2, 11)^c^
Percent of patients with antiemetic medication use	65.1	69.8	66.5	59.8
Days of antiemetic medication use	2 (1, 4)	2 (1, 5)	2 (1, 4)	2 (1, 4)^c^
Inpatient readmission within 30 days, for discharged patients (n=4709)	857 (18.2)	236 (11.6)	93 (15.3)^b^	528 (25.6)^c^

Values are mean (SD) or median (25th, 75th percentile) for continuous factors and % or n (%) for categorical factors. Nutrition utilization and billing were missing on n=16-24 of patients. Abbreviations: EN, enteral nutrition; HCRU, healthcare resource utilization; ICU, intensive care unit; IP, IMPACT® Peptide 1.5; LOS, length of stay; PC, Pivot® 1.5 Cal; StdHP, standard high-protein tube feeding products. ^a^*P*.01 for IP vs PC or IP vs StdHP. ^b^*P*.05 for IP vs PC or IP vs StdHP. ^c^*P*.001 for IP vs PC or IP vs StdHP.

**Figure 2.**
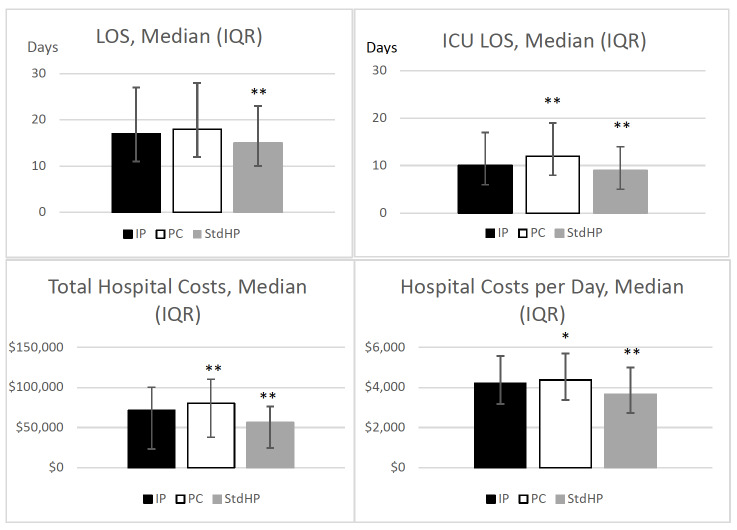
Healthcare Resource Utilization Comparisons of EN Groups^a^ Abbreviations: EN, enteral nutrition; HCRU, healthcare resource utilization; ICU, intensive care unit; IP, IMPACT® Peptide 1.5; IQR, interquartile range (ie, error bars); LOS, length of stay; PC, Pivot® 1.5 Cal; StdHP, standard high-protein tube feeding products. ^a^Results from unadjusted analyses. *P.05 for IP vs PC. **P.001 for IP vs PC or IP vs StdHP.

**Length of stay**: Length of stay was similar between IM cohorts (Figure 2); however, unadjusted results revealed patients receiving IP had higher median LOS than patients receiving StdHP (17 vs 15 days, *P*.001). For patients receiving IP, the median ICU LOS was lower compared with patients receiving PC (10 vs 12 days, *P*.001), but higher compared with patients receiving StdHP (10 vs 9 days, *P*.001).

**Medication use**: During hospitalization, 97.5% of patients were prescribed antibiotic, antiemetic, and/or antidiarrheal medications. The IP cohort had a significantly higher median of antibiotic days than the StdHP cohort (19 vs 17, *P*.001), but antibiotics days did not differ from the PC cohort. For patients on antiemetic medications, the median number of days billed was 2 for all cohorts. However, 69% of patients in the IM cohorts received an antiemetic compared with 60% of patients receiving StdHP (*P*.001 vs IP). Patients who received IP had a higher median number of antidiarrheal medication days than PC (9 vs 3, *P*.001) and StdHP (9 vs 5, *P*.001).

**Readmissions**: Within 30 days of discharge, the percentage of inpatient readmissions was lowest in patients receiving IP (11.6%) compared with patients receiving PC (15.3%, *P*=.014) and StdHP (25.6%, *P*.001).

### Cost Comparisons of EN Groups

**Total and daily cost**: Patients who received IP had a lower median total cost ($71 196 vs $80 696, *P*.001) and median total cost per day ($4208 vs $4373, *P*=.019) than patients who received PC. However, these costs were higher for IM groups compared with StdHP (Figure 2).

**Nutrition cost**: Patients who received IP had a lower median nutrition cost ($109 vs $248, *P*.001) than patients who received PC. Compared with StdHP, the cost for IM products was higher (Table 3).

**ICU stay cost**: Patients who received IP had a lower median cost of ICU stay compared with patients receiving PC ($22 687 vs $24 941, *P*=.001). However, the median costs of ICU stay were higher for IP compared with patients receiving StdHP ($22 687 vs $17 826, *P*.001).

**Medication cost**: Median total cost of antibiotic, antidiarrheal, and antiemetic medications did not differ between IM formulas. However, medication cost was higher overall for patients receiving IP compared with StdHP ($591 vs $416, *P*.001). Cost of antibiotics reflected the days of antibiotic use with no significant difference in the IM cohorts and lesser cost for the StdHP cohort. Although days of antiemetic medications did not differ across groups, the StdHP group registered the lowest cost for them. In a reversal of utilization results, the median cost per day of antidiarrheal medication was lower for IP compared with PC and StdHP groups, respectively.

### Total Cost per Day of EN Groups

**Figure 3.**
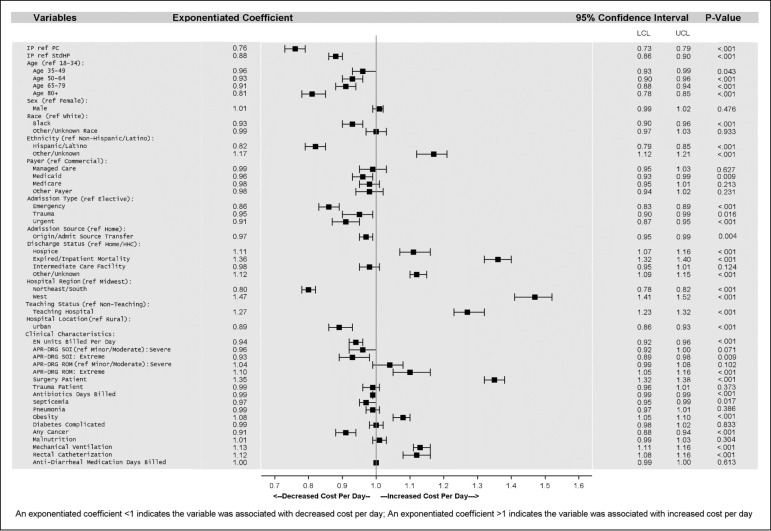
Multivariable Regression Estimates For Total Cost per Day *Note*: After adjustment for covariates, total cost per day for patients receiving IP was 24% lower compared with patients receiving PC and 12% lower compared with patients receiving StdHP. Abbreviations: APR-DRG, All Patient Refined Diagnosis Related Groups; EN, enteral nutrition; HHC, home healthcare; IP, IMPACT® Peptide 1.5; LCL, lower confidence limit; PC, Pivot® 1.5 Cal; ref, reference category; ROM, risk of mortality; SOI, severity of illness; StdHP, standard high-protein tube feeding products; UCL, upper confidence limit.

After adjustment for covariates, IP was associated with a 24% lower average total cost per day compared with patients receiving PC and 12% lower average total cost per day compared with patients receiving StdHP (Figure 3). Translated into approximated dollar values using least-square means in the generalized linear model, the estimated adjusted mean cost per day for the IP group was $4096 (95% CI: $3967, $4230) compared with $5381 (95% CI: $5159, $5612, *P*.001) in the PC group and compared with $4662 (95% CI: $4506, $4824, *P*.001) in the StdHP group, when all other variables in the model were held constant. Assessment of and correlations between covariates, tests of multicollinearity, and model fit statistics indicated that a sound multivariable model was utilized.

### Demographic and Other Factors Associated With Cost Per Day

In the multivariable model, after adjustment for other covariates, clinical factors and demographics significantly associated with lower cost per day included older age (reference, 18-34 years; age 80+ years, 19% lower; age 65-79 years, 9% lower), cancer diagnosis (9% lower), nonelective admission (emergency, 14% lower; trauma, 6% lower; urgent, 9% lower), and amount of EN units billed per day (6% lower). Demographic factors associated with lower cost per day in the adjusted model included race (reference, white; Black, 7% lower) and Hispanic/Latino ethnicity (18% lower), among others.

Holding other variables in the model constant, factors significantly associated with a higher cost per day in the multivariable model included surgery (35% higher), mechanical ventilation (13% higher), rectal catherization (12% higher), death at discharge (reference, discharge home; 36% higher), APR-DRG ROM extreme (reference, minor/moderate; 10% higher), and obesity diagnosis (8% higher).

## DISCUSSION

This study examined descriptors and outcomes associated with a large group of patients hospitalized with an ICU stay, who received 1 of 3 options of high-protein EN. Overall, patients received a median 7 L of EN across 7 days during a 16-day hospital stay with 10 days in the ICU. The many differences in clinical diagnoses, comorbidities, and HCRU variables made the adjustment for multiple potentially confounding variables necessary and critical to drawing conclusions.

Choice of high-protein EN for patients in the ICU has implications for HCRU and daily hospital costs. It is important to consider these correlations when comparing formula ingredients and per-unit costs. Among the EN products with added immunonutrients, IP emerged as the most cost-saving option in this retrospective study. Unadjusted analysis revealed that patients receiving IP reported lower total cost of hospitalization, cost per day, cost of ICU stay, and nutrition product cost compared with PC. These differences may be partially due to shorter ICU LOS, fewer comorbidities, and/or lesser illness severity for patients on IP compared with PC. Nonetheless, after adjustment for covariates, IP was associated with 24% lower average total cost per day compared with patients receiving PC. Additionally, IP was associated with 12% lower adjusted average total cost per day compared with patients receiving StdHP, for whom all cost/stay measures were lower than IP in unadjusted analyses. Fewer inpatient readmissions within 30 days for patients receiving IP, compared with patients receiving PC or StdHP, suggest the need for future evaluation to determine potential additional cost savings.

Previous health economic analyses of IM formulas have shown savings related to IM vs standard of care in major elective gastrointestinal surgery and trauma, and in a smaller population of elective surgery, trauma, and medical patients who may not have had an ICU stay.^10–12^ Consequently, the size and scope of this new analysis make an important contribution to understand the relationship between the cost of acute hospitalization and the choice of high-protein EN (IP vs PC vs StdHP).

When comparing IM formula groups, rectal catherization, pneumonia, and septicemia were lower for patients receiving IP compared with PC, although LOS and days of nutrition product use were similar between these groups. Frequency of pneumonia and septicemia were also lower for patients receiving IP compared with StdHP, for whom LOS and days of nutrition product use were less than IP. Interestingly, days of antidiarrheal medication use was higher for patients receiving IP compared with PC and StdHP, yet antidiarrheal cost per day was lower for IP vs PC or StdHP. Obtaining information on dose and type of anti-diarrheal prescribed was beyond the scope of this analysis, making these opposing results difficult to interpret. It is unfortunate that indicators of EN intolerance were not better captured in these cohorts, as previous retrospective data has shown a lesser frequency of diarrhea associated with IP when IP and PC were compared.^19^ Taken together, these factors suggest that before a rationale for formula composition differences can be formed in relationship to cost, an adjusted analysis of clinical outcomes is needed.

Malnutrition or undernutrition is a frequent problem in hospitalized patients, and the clinical diagnosis is associated with significant longer LOS and higher costs.^20–23^ By reducing LOS, lowering infection rates, and avoiding readmissions, early nutrition therapy targeting malnourished hospitalized patients suggests total healthcare cost savings.^16,24^ In the ICU, appropriate nutrition management is vital to avoid potential risks.^25^ Given the characteristics and comorbid conditions of our real-world sample, proper choice of high-protein EN is essential.

Limitations of this study, such as reliance on accurate and complete diagnostic/procedural coding and similar cost accounting methods between hospitals, are inherent to any administrative database. Additionally, since patients in the EN cohorts were not randomly assigned, there was potential selection bias because choice of formula may have been limited by hospital formulary, insurance payer, formula cost, clinician preference, and/or consideration of comorbidities. Although multivariable regression modeling was performed to control for bias, differences in unmeasured characteristics and endogenous heterogeneity may exist. Follow-up events that may have occurred outside the PHD were not captured. It must also be acknowledged that this work did not include a chart review, and therefore obtaining information on nutritional adequacy (formula administered in relationship to needs) was not included in this analysis.

Although great effort was made to control for potential confounders, it is important to acknowledge the IM cohorts and StdHP cohort had some significant differences. The StdHP group was older, had fewer surgeries, and had far fewer trauma patients. The IP and PC groups also had many more patients who needed tracheostomy, possibly related to trauma diagnoses, than the StdHP cohort. Thus, study results may not be generalizable to all critically ill patients, and differences in severity may have influenced the magnitude of associations. For these reasons, we suggest further adjusted analysis of clinical outcomes be limited to the IP and PC cohorts, which may allow correlations to be drawn in association with IM formula differences. Furthermore, propensity score matching may be helpful to address the differences between the cohorts.

## CONCLUSIONS

Using a select IM formula for critically ill patients may provide cost savings for the overall healthcare system. This study used real-world data to comprehensively examine descriptors and outcomes associated with use of 3 options for high-protein EN in patients hospitalized with an ICU stay. Of the 2 IM groups, IP was associated with significantly lower average total cost per day compared with use of PC, after controlling for demographic, visit, hospital, and clinical characteristics. Further, after controlling for these variables, the IP cohort was also associated with significantly lower average total cost per day compared with StdHP. Additional studies are required to corroborate the findings of this study; however, when comparing ingredient differences in high-protein EN formulations and product costs, these results show the importance of considering overall HCRU (eg, total cost per day) when evaluating potential cost saving for hospitalized patients who are critically ill.

### Disclosures

A.M.D., M.K.M., C.C.L., and K.A.T. are employed by Nestlé, M.C.B. is employed by University of Louisville Health and provided consulting services to Nestlé, and L.L.S., D.L.B., and Z.C. are employed by Premier Inc, which received funding from Nestlé to complete this research.
